# Indicators From China’s Listed Corporations on Corporate Financing Behavior and Policy-Related Risk

**DOI:** 10.3389/fpsyg.2022.930929

**Published:** 2022-08-18

**Authors:** Wenlong Zhang, Muhammad Arslan Ausaf, Junaid Jahangir

**Affiliations:** ^1^School of Finance, Shanxi University of Finance and Economics, Taiyuan, China; ^2^Department of Business Administration, Preston University, Islamabad, Pakistan

**Keywords:** corporate finance behavior, uncertainty, geopolitical risk, political risk, policy

## Abstract

This study explores corporate financing behavior regarding the company and country-level factors and risks associated with policy-related regulations. The study considers all three categories of risk: geopolitical risk, economic policy uncertainty, and political risk. In addition to this, we investigated how the links between diverse types of financing activities and the policy-related risks associated with them change depending on the type of financing strategy utilized (debt vs. equity). The study examined quarterly data from 2016Q1 to 2020Q3. EViews 12 is used for data analysis. Findings show financial restrictions, as well as inequities within the sector, have an impact on corporate investment while policy-related risks might impact a company’s financing selections. Compared to equity financing, debt financing is more susceptible to policy-related risk. According to the available information, features at the company and nation levels also impact corporate finance choices. Finally, firms that have little financial resources are more susceptible to the adverse effects of policy-related risk than industrial companies are. Managers, as well as governments, should utilize these insights to design economic strategies that are more successful in the future.

## Introduction

Some factors affect how firms are managed, how well they perform, and how the economy operates (Karpavičius and Yu, 2019). Several years have been spent investigating the elements that influence companies’ decisions to obtain financing (Lee et al., 2017a). It has been shown that firm-level characteristics such as size, profitability, cash flow, and growth potential impact financing behavior; however, it is unclear if these factors are the only ones that impact (Graham et al., 2015). From the perspective of macroeconomics, a government’s policies may significantly influence the organizational working and progressive climate. For example, monetary policy impacts the amount of money from outside sources and the amount that must be repaid to investors once investment projects are completed (Panousi and Papanikolaou, 2012). Because of contractionary monetary policy, the cost of borrowing increases. Discount rates higher than the market rate reduce investment rates, minimizing the requirement for outside money. We still do not understand a great deal regarding the influence of macroeconomic and institutional changes on elements at the nation level (Pindado et al., 2017). Our research attempts to determine whether factors at the national level impact the characteristics of a firm.

Because policy-related shocks are often cited as the primary source of uncertainty in the business environment, it is logical to question if this policy-related risk significantly impacts how firms decide to borrow capital in the first place (Dixit and Pindyck, 1994). It is unclear how policy-related risks impact the financial choices of corporations because of a lack of information. The economic policy uncertainty (EPU) model is named after the authors (Baker et al., 2016). The paper covers a wide variety of economic and policy risks; there has been greater recognition that geopolitical risk and uncertainty impact economic cycles and the performance of financial markets in recent years (Lee et al., 2019). Caldara and Iacoviello (2018) established a geopolitical risk index, and the International Country Risk Guide (ICRG) index assesses the risk associated with policymaking in various countries. We will be able to understand better how policy risk impacts business finance in this manner by using these new indexes.

Over several decades, the many types of research on how businesses generate new capital were concentrated on the capital markets of industrialized or developed countries in the Western hemisphere. For example, Baum et al. (2009) conducted research on non-financial businesses in the United States between 1993 and 2003. They discovered that uncertainty decreased the companies’ willingness to borrow money. According to the findings of research that Frank, and Goyal (2009) conducted on a substantial number of non-financial United States firms between the years 1950 and 2003, the level of corporate financing decreases with a company’s level of profitability while simultaneously increasing with the size of the business and the rate of inflation over time. Qiu and La (2010) analyzed the reasons that led to the considerable shift that occurred in the capital structure of Australian corporations between the years 1992 and 2006 and found that several factors were involved. They realized that an increase in their wages led to a reduction in their debt. On the other side, the transitory economy has not gotten much attention. Because of their government’s power and the political difficulties, they face, countries amid change have a substantial effect on the actions of businesses (Shleifer and Vishny, 1992).

**FIGURE 1:**
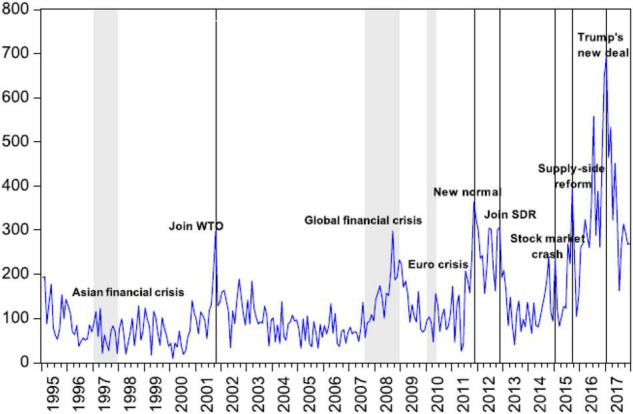
Variations in the EPU index for China.

Our research in China differs from other countries. Primarily, due to the nation’s expanding economy, the country’s financial markets, financial institutions, and commercial practices have grown in importance across the globe (Jiang et al., 2017). Second, it has been widely documented that China has transitioned from a centrally planned economy to a market-based economy in recent years (Wang et al., 2014). China is still a centrally planned economy, even though it is not as active as it once was. Controlling and influencing economic activity are accomplished via the deployment of central policies. Furthermore, China’s capital market has more defects and expenses, making it difficult for firms to make financial judgments. Resultantly, China is an excellent location to assess the impact of policy risk on financial behavior. When significant economic and political shifts in China, the EPU gains importance (Figure 1). We must learn more about how China’s EPU impacts the financing operations of its firms since the findings may have ramifications for other nations that are in the process of becoming more economically stable.

In this article, we examine how the company and country-level characteristics, and policy-related risks influence corporate financing choices using quarterly data spanning the period 2016Q1 to 2020Q3, focusing on the United States. There are four primary points to this research. This section will examine how policy-related risks such as policy uncertainty, political risk, and geopolitical risk influence corporate finance when a shock is produced by a change in government policy first. Multidimensional measurements provide for a more thorough evaluation than a single statistic alone might provide. Because China’s financial markets are expanding at such a rapid pace, our study helps to fill in some of the gaps in the available information on how rapidly they are growing. It is essential to notice that our results significantly affect nations not as developed as they should be. Third, we investigate how policy-related risks influence how corporations raise debt and equity financing funds. We split the sample into groups with varying degrees of financial constraint and enterprises to evaluate whether the various firm and business characteristics influence corporate financing choices.

The authors demonstrate that variables at both the business and the national level influence how cooperatives choose to fund one another, just as they did in the old capital structure theory. In addition to economic, geopolitical, and political risk, uncertainty significantly influences how organizations choose to seek capital. Moreover, it demonstrates that policy-related risk has a unique effect on debt financing than equity financing. Additionally, when the company and sector characteristics are considered, policy-related risk has a more substantial adverse influence on firm financing for organizations that have much money to spend and manufacturing companies.

## Literature Review and Theoretical Foundations

### Corporate Financing Theories

There has been a terrific agreement of theoretical and empirical research on the factors that influence whether firms opt to borrow money. The outcomes have been varied like in Trade-off theory (Miller, 1977; Myers, 1984); Pecking order theory (Jensen and Meckling, 1976; Miller, 1977). Academics and entrepreneurs have recognized the firm size, development potential, and profits as influencing factors alike for a long time. As per the pecking order theory, big organizations are less likely to suffer from information asymmetry than smaller organizations because they have more employees. the theory also supports that organizations that are undergoing elevated levels of development but do not have sufficient resources are more prone to take out loans than other sorts of companies (Kayo and Kimura, 2011).Also, the successful organizations are more inclined to spend their own money to decrease the danger of borrowing money in the future. According to the initial findings, the size of a company has a beneficial impact on corporate finance (Islam and Khandaker, 2015; Pindado et al., 2017; Karpavičius and Yu, 2019).

For instance, the agency theory suggests that taking on debt is a savvy method to prevent management and investors from engaging in conflict. It may be accomplished by using debt as a buffer between the two groups. Companies that do not have many prospects for growth or investment may be more prone to have unethical conflicts and adversarial selection than companies with these kinds of opportunities. If a firm decides to take on debt, there is a possibility that it will be able to reduce the total amount of money that it would be required to spend if it did not employ this method of financing (Kayo and Kimura, 2011). Consequently, growth may be positively or negatively related to corporate finance, depending on the context of the relationship being considered. In the actual world, the monetary value of the growth potential has not yet been quantified to anyone’s satisfaction. Some individuals are under the impression that the two concepts are unfavorably connected; however, this is not the case (Chang et al., 2019; Karpavičius and Yu, 2019; Liu and Zhang, 2019).

According to the trade-off theory, high-level-profitability enterprises are less likely to go bankrupt due to tax breaks and incentives. It implies that they will be able to borrow more money since they will be less likely to default on their debt (Frank and Goyal, 2003). Numerous studies have shown that money and profits do not go hand in hand (Rajan and Zingales, 1995; Dang et al., 2018; Chang et al., 2019). In addition to above studies, it is agreed that factors at the national level, such as the macroeconomic situation and the uncertainty that comes with it, are critical considerations for firms when determining the optimal capital structure. Businesses operating at various periods of the economic cycle, on the other hand, may have a variety of options for raising capital (Halling et al., 2016), (Paulson et al., 2021). Researcher utilize macroeconomic factors to demonstrate how changes in the economy impact the choices made by firms when it comes to financing (Dewally and Shao, 2014; Karpavičius and Yu, 2017). Among other things, Frank and Goyal (2009) investigated how non-financial enterprises employed leverage from the 1950s through the 2003s. They looked at how many different elements were at play. According to their findings, the amount of debt a firm owes grows with both GDP growth and expected inflation rates. Previous studies examined how bank lending changed throughout the global financial predicament of 2007–2009 and discovered that GDP growth had a significant influence on bank lending during this period (Dewally and Shao, 2014). According to Baum et al. (2009), macroeconomic and firm-specific uncertainty damage non-financial enterprises in the United States.

To summarize, the present study placed a strong focus on company characteristics as determinants in corporate finance, in the light of previous studies. Although the data suggests otherwise, there is no evidence that macroeconomic uncertainty and company debt are related. Therefore, policy-induced uncertainty has received extraordinarily little attention yet. As far as we are aware, there is not much research that examines how policy-induced shocks influence the financing choices of businesses. This article discusses how elements at the corporate and national levels and public policy considerations influence business financing and lending decisions. This study fills the gaps in the literature and explains prior findings that were inconsistent with one another.

### Policy-Related Risk Impact

The researchers are concerned about how policy-related risks such as policy uncertainty, political risk, and geopolitical risk may influence the real economy (Lee and Lee, 2018; Zhang et al., 2019b). These risks are considered the highly significant factors affecting the business cycle, employment, and the overall economy. According to the fundamental options theory, the value of a waiting option improves due to changes in the market and uncertainty, which might cause a corporation to postpone its investment operations (Kang et al., 2014). When it comes to other financial choices, Francis et al. (2014) points out that political uncertainty may impact the cost of debt. Baum et al. (2006) discovered that delay concerning future economic circumstances impacts the amount of cash that enterprises seek to have on hand. As uncertainty increases in the corporate environment, managers will become more cautious and, as a result, will continue to follow the same cash management guidelines. According to Phan et al. (2019), organizations subject to a high-level degree of uncertainty regarding economic policy are more likely to hold more cash on hand. It is because people desire to be protected.

academics have, in general, paid less attention to how policy-related risk affects financing operations than how corporations determine the process of spending their money. Baum et al. (2009) concluded that uncertainty does play a part in the decision-making process of businesses after researching to analyze the impact that macroeconomic uncertainty plays in the decision-making process of firms. Earlier studies have indicated that economic and policy uncertainties make it more difficult to draw money from other nations. These concerns are expected to make it more difficult to attract money from China. The fact that there are a more considerable number of unknowns is regarded to be the root cause of these challenges. The acquisition of financial resources from many foreign nations will become increasingly difficult. The equity risk premium (P′astor and Veronesi, 2013b), debt costs (Francis et al., 2014), and default risk (P′astor and Veronesi, 2013a) all increased to more significant levels as a direct result of this (Gilchrist et al., 2014). Lee et al. (2017b) present evidence that the degree of policy uncertainty influences how persons working in the banking business in the United States use leverage. Consequently, the risk associated with the policy is likely to have a significant effect on the decisions that businesses make about the investments they make with their money. We have investigated how policy uncertainty, political risk, and geopolitical risk affect the performance of non-financial businesses. Our analysis, which focuses on how these factors come into play, adds to the existing body of knowledge by examining how policy-related risks influence the funding decisions of non-financial firms in China.

## Methodology

In principle, policy shocks have always been the most significant factor impacting the economy. They affect both the supply and demand sides of the equation. External uncertainty increases information disproportionateness, future cash flow uncertainty, and default risk on the supply side resulting in a credit crisis (Zhang et al., 2015). Businesses confronted with elevated levels of peripheral uncertainty must keep their finances lean to cope with the detrimental effects of such uncertainty on operations (Graham and Harvey, 2001). When there is an immense agreement of uncertainty concerning how much money a business will produce in the future, the corporation will reduce the amount of money it wishes to borrow to compensate. It will assist in mitigating financial risk and keeping the cost of borrowing and bankruptcy at a bare essential minimum. According to P′astor and Veronesi (2013a), businesses may be less willing to invest due to policy-related risk since they may not be able to recover their investment costs. As a result, they are less likely to need funding.

Considering those mentioned earlier, empirical evaluation of the policy-related risk and business finance link is necessary. Our study is based on the models developed by Julio and Yook (2012) and Lee et al. (2017a). Firm finance is influenced by cash flow, growth potential, company size, and profitability. Researcher examined this problem considered these factors (Dang et al., 2018).

The standard models to be used are extensions of panel regressions, which are ubiquitous in the finance field and are used in this study.


(1) $${\text{AF}}_{\text{i},\text{t}}=\alpha_{\text{i}}+\beta_{1}{\text{PR}}_{\text{i},\text{t}-1}+\beta_{2}{\text{Cf}}_{\text{i},\text{t}-1}+\beta_{3}{\text{TQ}}_{\text{i},\text{t}}+\beta_{4}{\text{SG}}_{\text{i},\text{t}}+\gamma {\text{X}}_{\text{i},\text{t}-1}+\mu_{\text{i},\text{t}}$$

Equation 1, i is the cross-sectional unit, and t is the period. The dependent variable in this conventional regression is AF_*i,t*_. P R_*i,t*_ denotes policy-related risk metrics such as economic policy uncertainty, political risk indices, and geopolitical risk. CF_*i;t*_ is for cash flow, whereas TQ_*i,t*_ and SG_*i,t*_ stand for investment and growth prospects. The X_*i;t*_ controls include firm-specific kinds like size (SIZE) and profitability (PROFIT) (ROA). Finally, the unobserved firm-fixed impact is μ_*i,t*_.

Furthermore, we employed country-level variables as control variables to stipulate a complete picture of how firms choose to fund their operations. We accounted for GDP growth and inflation on the pattern of Frank and Goyal (2009). It resulted in the following modifications to the benchmark model.


(2) $${\text{AF}}_{\text{i},\text{t}}=\alpha_{\text{i}}+\beta_{1}PR_{\text{i},\text{t}-1}+\beta_{2}Cf_{\text{i},\text{t}-1}+\beta_{3}TQ_{\text{i},\text{t}}+\beta_{4}SG_{\text{i},\text{t}}+\gamma {\text{X}}_{\text{i},\text{t}-1}+\delta {\text{M}}_{\text{i}.\text{t}-1}+\mu_{\text{i},\text{t}}$$

Here, country-level considerations Mi;t includes the variation of inflation (INF)and the GDP growth rate (GDP).

### Description of Data

The China Stock Market & Accounting Research Database(CSMAR) database contains accounting statistics and other macroeconomic indicators. Table 1 lists the variables. We evaluated the short-term effects of policy-related risk using quarterly data from 2016Q1 through 2020Q3. EViews 12 is used for data analysis, which is updated available version. China’s Securities Regulatory Commission started demanding quarterly financial disclosures from all publicly listed enterprises in 2003, a move that continues today. The financial services industry and enterprises that get Special Treatment (ST) are not included in the sample. Financial variables are winorized at 1 percent in both tails to reduce the effect of outliers on the overall results. There are a total of 111,870 firm-quarters in the whole data collection.

**TABLE 1:** Variables’ list, definitions, and data resources.

Variable	Definition	Source
Actual financing (AF)	It is defined as Actual financing flows/total assets	China Stock Market & Accounting Research Database(CSMAR)
Debt-financing (DF)	Debt financing is defined as Debt financing/total assets	CSMAR
Equity-financing (EF)	Equity financing is defined as Equity financing/total assets	
Economic strategy uncertainty (ESU)	In evaluation of China’s economic policy uncertainty, a higher index indicates higher uncertainty.	Baker et al., 2016
Geopolitical consequence (GPC)	A higher index indicates higher risk in an assessment of geopolitical risk.	Caldara and Iacoviello, 2018
Political peril (POP)	An evaluation of political risk, with zero having been high and 100 having below.	China Stock Market & Accounting Research Database(CSMAR)
Cash flow (CF)	Net cash flow is defined as Net cash flow/total assets	China Stock Market & Accounting Research Database(CSMAR)
Tobin’s q (TQ)	An evaluation of investment prospects, proxies by the ratio of the market value of equity to the book value of total assets	China Stock Market & Accounting Research Database (CSMAR)
Sales growth (SG)	An evaluation of growth opportunity, proxies by the percentage difference in sales	China Stock Market & Accounting Research Database (CSMAR)
Firm size (SIZE)	Firm size means that the Natural logarithm of total assets	China Stock Market & Accounting Research Database (CSMAR)
Return on assets (ROA)	Return on assets represents Net profits/total assets	China Stock Market & Accounting Research Database (CSMAR)
Inflation (INF)	Percentage difference in the consumer price index means inflation	China Stock Market & Accounting Research Database (CSMAR)
GDP growth (GDP)	GDP indicates as GDP growth rate	China Stock Market & Accounting Research Database (CSMAR)

The study used policy-related uncertainty, political, and geopolitical risk to proxy policy-related risk. The policy uncertainty index was calculated using the EPU score developed by Baker et al. (2016). Thirty-two Chinese newspapers were used to calculate the size of the newspaper-based policy uncertainty indices, which are updated monthly based on the newspapers’ economic, policy, and uncertainty content. According to Caldara and Iacoviello (2018) the newly established geopolitical risk index is an indicator of regional and global geopolitical risk. In terms of external global uncertainty, this metric encompasses a broad range of events such as terrorist attacks and other geopolitical issues (Akbar et al., 2022). An assessment of the economic and political stability of a country has been made. These indices, which provide extensive monthly evaluations, allow for more systematic data collection in empirical studies since they permit more complete monthly reviews (Hoti, 2005; Lee et al., 2017a, 2019). Because policy-related risk is a complicated concept that a single proxy cannot represent, these indicators allow for a more comprehensive assessment. These indices are excellent policy-related risk proxies due to the factors outlined above (Lee et al., 2019).

Table 2 contains a summary of our descriptive data. Chinese debt is the country’s principal source of foreign finance, as shown by the average debt to equity ratios of 0.267 and 0.033, respectively. While economic policy uncertainty is highly unpredictable, political risk is the minimum unexpected. Another control variable that is compatible with past research is average cash flow. Tobin’s q (TQ) is another control variable consistent with previous research (Yang et al., 2019). The average logarithm of total assets is 21.626, a positive number (Yang et al., 2019). The relative risk (ROR) is 0.029, comparable to prior China research (Liu and Zhang, 2019).

**TABLE 2:** Statistics.

Variables	Mean	SD	Min	Max	Observations
AF	0.2153	0.2633	–0.1609	14.3392	112,970
(DF)	0.2767	0.2576	3.93E-09	1.2756	94,481
(EF)	0.0318	0.1168	–0.1381	2.4671	112,970
(EPU)	5.0072	0.5889	3.9059	6.3455	113,052
(GPR)	4.5782	0.1509	4.2346	5.2282	113,052
(POL)	4.1354	0.0746	4.0113	4.2656	113,052
(CF)	0.0138	0.0687	–0.1668	0.2213	108,622
(T.Q.)	2.1240	1.7841	0.2098	11.7637	104,161
(SG)	0.1234	0.5506	–0.8482	4.7730	104,669
(SIZE)	21.6256	1.1773	19.0132	25.6682	108,642
(ROA)	0.0192	0.0415	–0.0793	0.1690	108,582
(GDP)	0.2527	0.6924	–0.7606	1.1588	111,852
(INF)	–1.23E–04	0.0202	–0.0514	0.0405	111,852

## Empirical Results

### Association Between Firm Financing and Policy-Related Risk

For the first time, we estimated the yardstick regression utilizing just the risk index and a few critical financial variables from the literature, such as cash flow, Tobin’s q, sales growth, company size, and profitability. The estimates for the dependent variable, actual funding, using the panel fixed effect model are shown in Table 3. The results of the baseline requirements are displayed in columns (1)–(3), with standard errors clustered at the firm level, indicating that the baseline criteria were met. Company financing activities are significantly impacted by the uncertainty of economic policy and geopolitical risk, which means that the more uncertain monetary policy and geopolitical risk, the lower the motivation to finance. There is a significant correlation between financial activity and POL regarding political risk. Increasing POL (political stability) lowers political risk and, as a result, improves the amount of finance available. Alternatively, as political risk rises, the cost of commercial borrowing increases. Uncertainty and risk are considered economical and financial activity inhibitors. Businesses become more circumspect when selecting financing choices and they borrow less during periods of policy uncertainty to avoid surprise financial losses (Phan et al., 2019). Uncertainty raises borrowing rates, causing financial limits to be imposed on businesses (Lee et al., 2019; Liu and Zhang, 2019).

**TABLE 3:** Actual financing results.

Variables	(1)	(2)	(3)	(4)	(5)	(6)
(EPU)	–0.022*** (–0.003)			–0.011*** (–4.483)		
(GPR)		–0.229*** (–24.979)			–0.265*** (–25.127)	
(POL)			0.184*** (5.200)			0.188*** (5.135)
(CF)	0.142*** (5.219)	0.066*** (2.8677)	0.137*** (4.813)	0.054** (2.412)	–0.005 (–0.145)	0.061** (2.433)
(TQ)	0.008*** (4.219)	0.007*** (3.351)	0.012*** (5.381)	0.012*** (4.459)	0.011*** (4.432)	0.014*** (6.177)
(SG)	0.059*** (19.016)	0.056*** (18.177)	0.059*** (18.747)	0.059*** (21.573)	0.054*** (19.387)	0.067*** (21.468)
(SIZE)	0.021*** (7.543)	0.021*** (8.465)	0.027*** (6.861)	0.016*** (5.154)	0.027*** (10.376)	0.027*** (6.757)
(ROA)	–0.0687* (–1.7211)	–0.212*** (–5.210)	–0.124*** (–2.566)	–0.452*** (–9.753)	–0.567*** (–11.337)	–0.524*** (–12.221)
(GDP)				0.044*** (44.714)	0.054*** (44.662)	0.055*** (47.676)
(INF)				0.378*** (4.783)	–0.512*** (–7.268)	0.425*** (5.254)
Firm FE	YES	YES	YES	YES	YES	YES
Cluster	YES	YES	YES	YES	YES	YES
Housman	0	0	0	0	0	0
F-test	0	0	0	0	0	0
Adjusted R^2^	0.145	0.156	0.145	0.161	0.173	0.162
Observations	92,620	92,710	93,720	91,717	91,717	91,717

Robust t-values collected at the corporation level are stated in digressions. A constant term is contained but not stated to conserve space. ***, **, and * suggest statistical impact at the 1, 5, and 10% points, correspondingly.

The findings show that most of the control variables are statistically significant in their respective groups. Liu and Zhang (2019) and Dang et al. (2018) both report positive correlation coefficients, demonstrating that financing activity increases because of improved cash flows (2019). An explanation for this finding is the risk-taking behavior of businesses themselves. Because they have a more excellent borrowing capability, organizations with high cash flow are more inclined to borrow money. The higher the investment possibility or growth potential, the greater the motivation for a corporation to invest as a result of this (Gupta, 1969; Chang et al., 2019; Liu and Zhang, 2019; Zhao et al., 2021).

The SIZE coefficient is positive, showing that the size of a corporation influences the operations of financing organizations. These results are supported by several studies, including Karpavičius and Yu (2019). Larger companies may be able to get financing from outside sources more readily and may be able to take advantage of higher tax benefits (Lee et al., 2017a). Several studies, including Kayo and Kimura (2011), Dang et al. (2018), Pindado et al. (2017) support the pecking order theory’s prediction that return on assets is negatively related to company funding. A higher level of profitability reduces the requirement for outside financing in this area.

These settings may be problematic because they solely record firm-level features and do not consider macroeconomic factors. To solve this, two new country-level indicators, inflation, and GDP growth, have been introduced to serve as proxies for the present state of the economy. As shown in Columns 4–6, these expanded requirements simply continue our baseline definition. Neither the three policy-related hazards nor any other control variables had any impact. Nevertheless, they are favorable since they demonstrate that financial activity grows parallel with economic progress and inflation. Strong GDP growth and inflation imply favorable economic circumstances; firms anticipate making more money and incurring more debt due to the robust growth and inflation. Consider the fact that when we integrate country-level variables into our model, the corrected R^2^ values improve by around 2%. Findings imply that the features of a nation influence how businesses fund themselves.

### Debt Financing and Equity Financing

Next, we examined how firm-level characteristics, country-level factors, and economic policy uncertainties affect future debt and equity financing choices. Tables 4, 5 provide the estimated findings for the panel fixed-effect model. According to the table, the criteria results are in Columns (1)–(3), while the higher specifications are in Columns (4)–(6). While these variables affect debt and equity financing, their impact varies depending on the investment type. The effects of policy risks, growth prospects, and economic circumstances are like previous research. Statistics show that monetary policy uncertainty, political risk, and geopolitical risk hinder debt funding more than equity financing. Debt financing has more severe policy-related risks than equity financing, as seen by the early coefficients (debt) being more significant than later coefficients (equity). Bank loans are China’s primary foreign finance source, so these results appear plausible (Deng et al., 2013). The negative implications of uncertainty on economic activity have a more significant impact on financial institutions, which will impact the debt operations of enterprises because of the uncertainty.

**TABLE 4:** Assessed outcomes for debt financing.

Variable	(1)	(2)	(3)	(4)	(5)	(6)
(EPU)	–0.025*** (–13.783)			–0.016*** (–6.456)		
(GPR)		–0.103*** (–26.678)			–0.253*** (–31.478)	
(POL)			0.346*** (11.267)			0.367*** (12.897)
(CF)	0.647*** (22.545)	0.667*** (22.445)	0.623*** (25.467)	0.592*** (24.187)	0.509*** (21.645)	0.589*** (23.765)
(TQ)	–0.017*** (–15.156)	–0.016*** (–17.923)	–0.013*** (–13.576)	–0.014*** (–12.445)	–0.015*** (–12.430)	–0.007*** (–6.123)
(SG)	0.028*** (12.432)	0.023*** (13.889)	0.025*** (12.912)	0.036*** (19.567)	0.033*** (15.468)	0.036*** (16.034)
(SIZE)	0.007** (2.490)	0.006* (1.987)	0.025*** (6.561)	0.004 (0.976)	0.011*** (3.565)	0.027*** (6.767)
(ROA)	–0.718*** (–15.822)	–0.845*** (–18.887)	–0.765*** (–19.342)	–1.245*** (–26.514)	–1.234*** (–25.979)	–1.318*** (–26.712)
(GDP)				0.071*** (62.456)	0.068*** (62.981)	0.072*** (65.681)
(INF)				–0.153*** (–3.427)	–0.856*** (–13.835)	–0.138*** (–2.897)
Firm FE	YES	YES	YES	YES	YES	YES
Cluster	YES	YES	YES	YES	YES	YES
Housman	0	0	0	0	0	0
F-test	0	0	0	0	0	0
Adjusted R^2^	0.361	0.37	0.383	0.418	0.419	0.409
Observations	82,028	82,028	82,028	81,076	81,076	81,076

***Indicates the level of significance. *, **, and *** suggest statistical impact at the 1, 5, and 10% points, correspondingly.

**TABLE 5:** Assessed outcomes for equity financing.

Variable	(1)	(2)	(3)	(4)	(5)	(6)
EPU	–0.005*** (–8.067)			–0.003*** (–4.361)		
GPR		–0.058*** (–19.431)			–0.074*** (–21.051)	
POL			0.052*** (6.931)			0.048*** (6.467)
CF	–0.010 (–1.313)	–0.025*** (–3.215)	–0.011 (–1.479)	–0.015* (–1.902)	–0.033*** (–4.245)	–0.016** (–2.055)
TQ	0.004*** (8.535)	0.003*** (7.757)	0.004*** (9.352)	0.003*** (7.691)	0.003*** (7.590)	0.004*** (8.645)
SG	0.008*** (15.823)	0.008*** (14.729)	0.008*** (15.573)	0.009*** (16.927)	0.008*** (15.253)	0.009*** (16.840)
SIZE	0.002*** (3.744)	0.003*** (4.987)	0.005*** (5.569)	0.001** (2.138)	0.005*** (7.951)	0.004*** (5.205)
ROA	0.090*** (8.714)	0.052*** (4.935)	0.081*** (7.881)	0.051*** (4.657)	0.020* (1.767)	0.038*** (3.529)
GDP				0.006*** (22.579)	0.005*** (21.874)	0.006*** (24.279)
INF				0.285*** (10.994)	0.042* (1.857)	0.296*** (11.241)
Firm FE	YES	YES	YES	YES	YES	YES
Cluster	YES	YES	YES	YES	YES	YES
Housman	0	0	0	0	0	0
F-test	0	0	0	0	0	0
Adjusted R^2^	0.113	0.122	0.113	0.116	0.127	0.117
Observations	93720	93720	93720	92707	92707	92707

***Indicates the level of significance. *, **, and *** suggest statistical impact at the 1, 5, and 10% points, correspondingly.

Moreover, we looked at the differences between debt and equity financing results to find if there are any patterns. According to the findings concerning the effect of cash flow revealed that it is favorably related to debt financing but adversely connected with equity financing. These findings provide credence to the idea of a pecking order. Companies with substantial cash reserves would prefer to use debt financing instead of equity financing since equity financing is more costly than debt financing. Specifically, our study shows that T.Q. has a detrimental influence on debt financing while positively impacting equity financing in terms of investment possibilities. Management, shareholders, and debt holders may be experiencing agency challenges, which might explain these findings. For example, when a firm’s investment opportunities are restricted, borrowing money may help reduce the cost of agency between management and their shareholders. The agency’s price may be reduced when stock is used instead of cash to transfer ownership between shareholders and debt holders. The return on assets (ROA) is inversely proportional to equity funding received in terms of profitability. The fact that successful companies have an easier time accessing the stock markets is one probable reason. The findings indicate that the inflation harms debt financing. It increases the cost of borrowing, reducing the amount of debt being used.

### Robustness Check

Table 6 shows the main findings’ robustness. We started by testing our results with a variety of companies and sectors. Financial constraints have been demonstrated to impact firm investment (Zhang et al., 2019a), cash holdings (Silva, 2019), and financing behavior (Silva, 2019; Zhang et al., 2019b). We separated the sample into two sub-samples depending on the dividend payout ratio to examine how financial constraints impact the link between policy-related risk and corporate finance. Although the impact on low-financial-constrained firms is unknown, policy-related risks significantly impact corporate financing decisions. Our results are unsurprising, given that high-financial-restricted businesses often lack internal liquidity and access to capital markets (Phan et al., 2019).

**TABLE 6:** Robust checks: low financially constricted and high financially constricted firms.

Variable	Low financially constrained			High financially constrained		
	(1)	(2)	(3)	(4)	(5)	(6)
EPU	0.024*** (7.218)			–0.017 (–5.267)		
GPR		–0.170*** (–10.898)			–0.334*** (–21.147)	
POL			0.026 (0.526)			0.309*** (5.301)
CF	–0.034 (–1.009)	–0.052 (–1.552)	–0.023 (–0.701)	0.228*** (5.471)	0.123*** (2.962)	0.225*** (5.426)
TQ	0.013*** (3.461)	0.014*** (3.726)	0.015*** (4.285)	0.009*** (3.401)	0.008*** (3.114)	0.012*** (4.512)
SG	0.055*** (16.828)	0.053*** (16.370)	0.055*** (16.892)	0.056*** (11.005)	0.050*** (10.026)	0.055*** (10.889)
SIZE	0.027*** (6.676)	0.040*** (10.226)	0.036*** (6.355)	–0.005 (–1.092)	0.016*** (3.954)	0.014** (2.334)
ROA	–0.336*** (–5.497)	–0.380*** (–6.236)	–0.335*** (–5.535)	–0.788*** (–10.649)	–1.052*** (–14.075)	–0.848*** (–11.654)
GDP	0.057*** (36.742)	0.054*** (35.129)	0.056*** (37.698)	0.051*** (29.215)	0.051*** (30.812)	0.052*** (31.156)
INF	0.244** (2.556)	–0.591*** (–6.829)	–0.005 (–0.056)	0.988*** (6.917)	–0.020 (–0.152)	1.088*** (7.523)
Firm FE	YES	YES	YES	YES	YES	YES
Cluster	YES	YES	YES	YES	YES	YES
Housman	0	0	0	0	0	0
F-test	0	0	0	0	0	0
Adjusted R^2^	0.167	0.169	0.166	0.248	0.268	0.249
Observations	52,422	52,422	52,422	38,237	38,237	38,237

***Indicates the level of significance. *, **, and *** suggest statistical impact at the 1, 5, and 10% points, correspondingly.

Manufacturing firms have a different financial structure and market competitiveness than non-manufacturing firms. According to Firth et al. (2012) and Jiang et al. (2015), manufacturing enterprises in China invest more than other businesses (2015). In this case, we divided the sample into two parts: manufacturing and non-manufacturing. Our findings show that policy-related risks harm manufacturing and non-manufacturing firms. Manufacturing firms, on the other hand, are more relevant and intense, results shown in Table 7.

**TABLE 7:** Robust checks: manufacturing and non-manufacturing firms.

Variable	Manufacturing			Non-manufacturing		
	(1)	(2)	(3)	(4)	(5)	(6)
EPU	–0.010*** (–3.551)			–0.006 (–1.613)		
GPR		–0.293*** (–23.702)			–0.229*** (–11.237)	
POL			0.224*** (4.652)			0.112 (1.594)
CF	0.105*** (3.073)	0.031 (0.914)	0.103*** (3.031)	0.022 (0.586)	–0.033 (–0.908)	0.017 (0.468)
TQ	0.010*** (4.004)	0.009*** (3.852)	0.013*** (5.350)	0.014*** (2.802)	0.014*** (2.637)	0.015*** (3.401)
SG	0.071*** (14.211)	0.066*** (13.497)	0.070*** (14.077)	0.046*** (15.869)	0.042*** (15.016)	0.046*** (15.864)
SIZE	0.011*** (2.840)	0.026*** (7.235)	0.027*** (5.281)	0.015*** (3.322)	0.026*** (5.936)	0.022*** (2.976)
ROA	–0.390*** (–6.742)	–0.513*** (–8.839)	–0.457*** (–7.823)	–0.641*** (–9.079)	–0.736*** (–10.638)	–0.660*** (–9.718)
GDP	0.056*** (37.063)	0.054*** (36.974)	0.057*** (39.108)	0.053*** (26.837)	0.052*** (27.216)	0.054*** (29.104)
INF	0.371*** (3.810)	–0.606*** (–6.539)	0.383*** (3.926)	0.440*** (3.389)	–0.327*** (–3.007)	0.467*** (3.554)
Firm FE	YES	YES	YES	YES	YES	YES
Cluster	YES	YES	YES	YES	YES	YES
Housman	0	0	0	0	0	0
F-test	0	0	0	0	0	0
Adjusted R^2^	0.189	0.203	0.19	–0.158	0.165	0.188
Observations	60,269	60,269	60,269	32,740	32,740	32,740

***Indicates the level of significance. *, **, and *** suggest statistical impact at the 1, 5, and 10% points, correspondingly.

Table 8 summarizes the results of the real estate, loan, and equity robustness tests. Columns 1–3 examine the influence of political stability, whereas Columns 4 to 6 examine the function of law and order. The GS and LO coefficients are always positive, regardless of the funding method. As a result, corporations are less motivated to invest when there are more legal and regulatory issues. Policy-related risks affect debt financing more than equity financing. It confirms our first assumptions.

**TABLE 8:** Robust checks: two alternate policy-related proxies.

Variable	AF	DF	EF	AF	DF	EF
(G.S.)	0.121*** (6.371)	0.168*** (12.387)	0.024*** (5.270)			
(L.O.)				0.120*** (5.314)	0.197*** (9.739)	0.030*** (6.432)
(CF)	0.057** (2.236)	0.568*** (21.918)	–0.016** (–2.122)	0.055** (2.148)	0.565*** (21.760)	–0.017** (–2.206)
(TQ)	0.012*** (5.143)	– 0.011*** (10.166)	0.003*** (7.973)	0.011*** (5.052)		0.003*** (7.984)
(SG)	0.068*** (21.527)	0.036*** (17.667)	0.008*** (15.772)	0.057*** (20.410)	0.038*** (19.275)	0.008*** (15.781)
(SIZE)	0.025*** (6.219)	0.017*** (5.024)	0.001*** (3.727)	0.024*** (7.135)	0.018*** (5.339)	0.004*** (4.4589)
(ROA)	– 0.464*** (–11.521)	–1.251*** (–26.408)	0.046*** (4.197)	–0.486*** (–10.595)	–1.256*** (–26.515)	0.045*** (4.097)
(GDP)	0.056*** (49.731)	0.072*** (65.484)	0.005*** (23.221)	0.055*** (48.756)	0.071*** (66.383)	0.005*** (23.351)
(INF)	0.366*** (5.925)	–0.178*** (–2.750)	0.2778*** (12.167)	0.421*** (5.367)	–0.131** (–2.445)	0.287*** (12.611)
Firm FE	YES	YES	YES	YES	YES	YES
Cluster	YES	YES	YES	YES	YES	YES
Housman	0	0	0	0	0	0
F-test	0	0	0	0	0	0
Adjusted R2	0.172	0.412	0.117	0.172	0.412	0.117
Observations	92,707	80,074	92,707	92,707	80,074	92,707

***Indicates the level of significance. *, **, and *** suggest statistical impact at the 1, 5, and 10% points, correspondingly.

## Conclusion and Implications

Since the 1970s, there has been a great deal of volatility resulting from policy uncertainty, which has piqued the interest of economists and investors everywhere. According to research, policy-related risks have a significant impact on the decisions made by businesses when it comes to making investments. Their effects on Chinese corporate finance, on the other hand, have been overlooked. This study extends previous research on business financing behavior by examining policy-related concerns such as economic policy uncertainty, political risk, and geopolitical risk, using quarterly data from 2016Q1 to 2020Q3. The data used in this study is from the first quarter of 2016 to the third quarter of 2020.

It is fair to hypothesize that companies exposed to an elevated level of policy-related risk will decrease their reliance on external investment. This theory can be supported by the evidence presented in this article. This conclusion reveals that policy-related risk has a considerable influence on corporate finance operations and how they are conducted. However, those in charge of policy at the government level are responsible for giving considerable thought to how changes to the policy impact the financial behavior of enterprises. However, policymakers should also focus on preserving the health of the macro economy, which should be their first goal, and primary concern. The well-being of the macro economy ought to be the top priority for those in charge of formulating economic policy. It has previously been demonstrated that the repercussions of the risk are far more severe in the case of debt financing than they are in the case of equity financing. These findings offer manager’s assistance in conceiving of and honing down on appropriate financial strategies and bringing to the attention of the public the requirement for policy change. In addition, the public has become more aware of the necessity of revising policies due to these discoveries. And because of these outcomes, the public has a heightened awareness of the importance of alterations to existing policies.

Research has concluded that decisions about corporate finance are impacted not only by elements specific to the firm but also by aspects specific to the nation in which the company is located. Other variables include a positive link between the financial state of a company and company-level characteristics such as cash flow, investment, growth, and scale. On the other hand, there is a negative association between profitability and the financial state. The study revealed that favorable conditions for business investment include a rising economy and a more considerable inflation rate. These are both distinctive features of a nation, contributing to favorable conditions for business investment. When making decisions about the firm’s finances, managers need to ensure that they take into consideration both the internal and external macroeconomic environments. We examined various business and industry characteristics to determine our findings’ validity. The study concludes, policy-related risks have equivalent inhibitory impacts on business investment, but their significance and severity differ. Both high-financial-constrained and industrial firms are more susceptible to policy-related risk, which impacts corporate finance. When policy-induced risks rise, governments should concentrate a more significant emphasis on the most vulnerable firms.

## Data Availability Statement

The original contributions presented in this study are included in the article/supplementary material, further inquiries can be directed to the corresponding author.

## Author Contributions

All authors listed have made a substantial, direct, and intellectual contribution to the work, and approved it for publication.
